# Uptake of COVID-19 and influenza vaccines in relation to preexisting chronic conditions in the European countries

**DOI:** 10.1186/s12877-023-04623-5

**Published:** 2024-01-12

**Authors:** Shangfeng Tang, Lu Ji, Ghose Bishwajit, Shuyan Guo

**Affiliations:** 1 School of Medicine and Health Management, Tongji Medical College of Huazhong University of Science and Technology, Wuhan, China; 2 Interdisciplinary School of Health Sciences, Faculty of Health Sciences, University of Ottawa, Ottawa, Ontario Canada; 3 National Institute of Hospital Administration, National Health Commission, Beijing, China

**Keywords:** COVID-19, Influenza, Vaccine, Chronic disease, Comorbidities, Vaccine hesitancy

## Abstract

**Background:**

The suboptimal uptake of COVID-19 and influenza vaccines among those with non-communicable chronic diseases is a public health concern, because it poses a higher risk of severe illness for individuals with underlying health conditions, emphasizing the need to address barriers to vaccination and ensure adequate protection for this vulnerable population. In the present study, we aimed to identify whether people with chronic illnesses are more likely to get vaccinated against COVID-19 and influenza in the European Union.

**Methods:**

Cross-sectional data on 49,253 men (n = 20,569) and women (n = 28,684) were obtained from the ninth round of the Survey of Health, Ageing and Retirement in Europe (June – August, 2021). The outcome variables were self-reported COVID-19 and influenza vaccine uptake status. The association between the uptake of the vaccines and six preexisting conditions including high blood pressure, high blood cholesterol, chronic lung disease, diabetes, chronic bronchitis, and asthma was estimated using binary logistic regression methods.

**Results:**

The vaccination coverage for COVID-19 ranged from close to 100% in Denmark (98.2%) and Malta (98.2%) to less than 50% in Bulgaria (19.1%) and Romania (32.7%). The countries with the highest percentage of participants with the influenza vaccine included Malta (66.7%), Spain (63.7%) and the Netherlands (62.5%), and those with the lowest percentage included Bulgaria (3.7%), Slovakia (5.8%) and Poland (9.2%). Participants with high blood pressure were 3% less likely [Risk difference (RD) = -0.03, 95% CI = -0.04, -0.03] to report taking COVID-19 and influenza [RD = -0.03, 95% CI= -0.04, -0.01] vaccine. Those with chronic lung disease were 4% less likely [RD = -0.04, 95% CI= -0.06, -0.03] to report taking COVID-19 and 2% less likely [RD= -0.02, 95% CI = -0.04, -0.01] to report taking influenza vaccine. Men and women with high blood pressure were 3% less likely to have reported taking both of the vaccines.

**Conclusions:**

Current findings indicate a suboptimal uptake of COVID-19 and influenza vaccines among adult men and women in the EU countries. Those with preexisting conditions, including high blood pressure and chronic lung disease are less likely to take the vaccines.

**Supplementary Information:**

The online version contains supplementary material available at 10.1186/s12877-023-04623-5.

## Introduction

COVID-19 has been a major global public health concern since its emergence in late 2019. It has spread rapidly throughout the world, causing huge disruption to the lives of millions of people. Its impact on global public health has been immense, with millions of fatalities and illnesses resulting from the virus, as well as a range of more long-term impacts including loss of livelihoods and economic hardship. [1] While the ongoing COVID-19 pandemic has been in the limelight of public health policies during the last three years, the influenza virus continues to be a significant health concern globally- a situation known as the dual burden of COVID-19 pandemic and a seasonal influenza epidemic. [1–3] Both COVID-19 and influenza are highly contagious respiratory illnesses that have been responsible for significant morbidity and mortality in recent years. COVID-19 is caused by a novel coronavirus, SARS-CoV-2, while influenza is caused by a group of orthomyxoviruses. It has been shown that COVID-19 may be more transmissible than influenza and can cause more severe disease in individuals who are immunocompromised or elderly. In 2017, Influenza lower respiratory tract infections was responsible for an estimated 145 000 deaths among all ages in 2017, with the mortality rate being highest among adults aged 70 years and higher. [4] In addition to being a major cause of respiratory mortality, influenza is associated with cardiovascular events, exacerbations of chronic underlying conditions, and increased risk for fatalities. [5] Most people generally recover within a week without requiring medical attention; however, influenza can lead to severe illness, hospitalization, and death, especially in older adults [4] who account for about 90% of the annual deaths occurring globally. [6].

Although the influenza vaccine is the most effective way to prevent influenza and its complications [7], the uptake of the vaccine is far below the WHO recommended level of 75% among individuals aged 65 years and higher. [8] WHO identified vaccine hesitancy (delay in acceptance or refusal of vaccines despite availability of vaccine services) as one of the 10 most serious threats to global health. [9] Previous studies have investigated the causes of vaccine non-uptake and hesitancy from various behavioral and social determinants of health perspectives. It has been shown that both COVID-19 and influenza disproportionately affect older individuals as well as those with preexisting conditions. [5, 10, 11] Evidence suggests that those who are less likely to take the seasonal influenza vaccine are usually those living with various chronic conditions such as asthma [12], cardiovascular diseases [13], COPD [14], and diabetes [15]. People who have COPD [16], diabetes [17] and psychiatric disorders [18] are also less likely to take COVID-19 vaccine. The non-uptake of vaccines for widespread health issues such COVID-19 and influenza is a major concern for healthcare systems, especially in countries with a high level of vaccine hesitancy, including the European Union. [19–21] As such, understanding the pattern of vaccination behavior among individuals with preexisting conditions is an urgent priority in the EU where older individuals account for about one-fifth of the entire population. [22] Currently, there is no comparative study on the prevalence of COVID-19 and influenza vaccination among older individuals in European countries. Therefore, in this study, we aimed to measure the prevalence of COVID-19 and influenza vaccination among adult individuals, and assessed whether the likelihood of vaccine uptake differs among individuals living with and without certain non-communicable chronic diseases.

## Methods

### Data source

Data for this study were derived from the ninth wave of the Survey of Health Ageing, and Retirement in Europe (SHARE). [23] The survey aims to provide a framework for researchers to better understand population ageing and covers most of the European Union and Israel. SHARE has been adopted as a model by a number of ageing surveys throughout the world and is harmonized with the US Health and Retirement Study (HRS) and the English Longitudinal Study of Ageing (ELSA). SHARE uses a standardized questionnaire that is translated by country teams into national languages (sometimes multiple languages) using an internet-based translation tool. The SHARE survey collects data on a diverse range of health and socioeconomic indicators among individuals including demographics, living conditions, income and employment status, mental health, satisfaction with health conditions and social networks. The ninth wave of the Survey included questions relevant to COVID-19, such as receiving support from social connections to obtain basic necessities. Data for this survey were collected via computer-assisted telephone interviews between June and August of 2021 among individuals aged 50 or older.

### Description of the variables

**Fig. 1:**
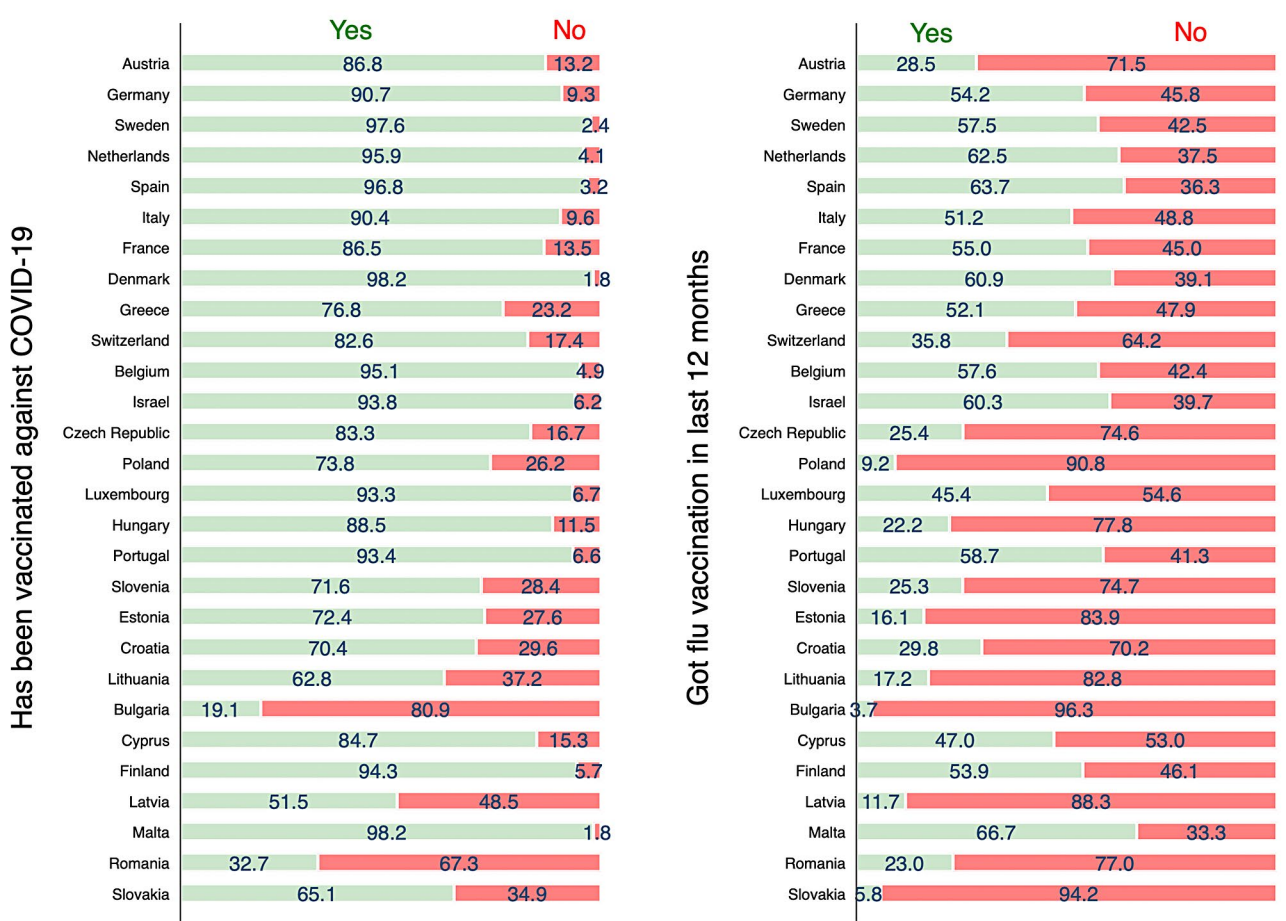
Percentage of participants with COVID-19 and influenza vaccines

The two outcome variables included COVID-19 (Has been vaccinated against COVID-19: Yes/No) and influenza vaccination (Got flu vaccination in last 12 months: Yes/No) status, which were assessed by participants’ own reports. Apart from the vaccination status, the survey collected data on several chronic conditions, including high blood pressure, high blood cholesterol, chronic lung disease, diabetes, chronic bronchitis, and asthma. These six preexisting conditions were the main explanatory variables for the purpose of this analysis, and they were assessed by the responses of the participants regarding the status of those conditions e.g. (has diabetes: yes/no), or taking medication for those conditions (taking medication for high blood pressure: yes/no). The analysis included several sociodemographic factors as well to adjust for their potentially confounding effects: current age (< 50 years/ 50–59 years/ 60–69 years/ 70–79 years/ 80–89 years/ >89 years); sex (Male/ Female); employment situation (Retired/ Employed/ Other); rating of self-reported health (Excellent/ Very good/ Good/ Fair/ Poor), and country of residence (appendix file ‘figure 1a’ for the list of countries).

### Data analysis

All statistical analyses were conducted using Stata 17 (Stata Corp., College Station, Texas). The dataset was first examined to ensure the target variables were labelled and calculated correctly and didn’t contain any outliers. Initial descriptive analyses included conducting bar charts to present the vaccination coverage for COVID-19 and influenza. This analysis was repeated to calculate the sex-stratified percentages for each country. The Risk differences of taking (1) COVID-19, (2) influenza and (3) both of the vaccines in relation to the six preexisting conditions were calculated using binary logistic regression methods. A positive RD value indicates a higher risk by the preexisting condition, whereas a negative one indicates a lower risk. Given the well-documented sex differences in vaccination behavior, [24, 25] the analysis was stratified by male and female participants for each outcome variable. The level of statistical significance for all associations was set at 0.05.

## Results

The sociodemographic characteristics of the sample population are presented in Table 1. The table shows that a greater percentage of the participants were aged between 60 and 69 years (36.2%), female (58.2%), retired (71.8%), and rated their health status as good (40.0%). More than half of the participants reported having hypertension (50%) and about two-fifth (40.2%) reported having high blood cholesterol. The percentage of participants with diabetes, bronchitis and asthma were 17.2%, 4.1% and 5.9%, respectively.

**Table 1:** Sample description

Age groups	Male (41.7%)	Female (58.2%)	Total
< 50 years	24 (0.1%)	160 (0.6%)	184 (0.4%)
50–59 years	1,479 (7.2%)	2,983 (10.4%)	4,462 (9.1%)
60–69 years	7,612 (37.0%)	10,231 (35.7%)	17,843 (36.2%)
70–79 years	7,613 (37.0%)	9,614 (33.5%)	17,227 (35.0%)
80–89 years	3,403 (16.5%)	4,845 (16.9%)	8,248 (16.7%)
> 89 years	438 (2.1%)	851 (3.0%)	1,289 (2.6%)
**Employment situation**			
Retired	15,798 (76.9%)	19,499 (68.1%)	35,297 (71.8%)
Employed	3,737 (18.2%)	4,715 (16.5%)	8,452 (17.2%)
Other	1,006 (4.9%)	4,429 (15.5%)	5,435 (11.1%)
**Rating of self-reported health**			
Excellent	976 (4.7%)	1,167 (4.1%)	2,143 (4.4%)
Very good	3,265 (15.9%)	4,383 (15.3%)	7,648 (15.5%)
Good	8,432 (41.0%)	11,274 (39.3%)	19,706 (40.0%)
Fair	5,989 (29.1%)	8,890 (31.0%)	14,879 (30.2%)
Poor	1,896 (9.2%)	2,946 (10.3%)	4,842 (9.8%)
**Diabetes**			
Yes	3,939 (19.2%)	4,511 (15.8%)	8,450 (17.2%)
No	16,556 (80.8%)	24,085 (84.2%)	40,641 (82.8%)
**Hypertension**			
Yes	10,362 (50.5%)	14,211 (49.7%)	24,573 (50.0%)
No	10,141 (49.5%)	14,383 (50.3%)	24,524 (50.0%)
**Chronic lung disease**			
Yes	1,490 (7.3%)	1,860 (6.5%)	3,350 (6.8%)
No	19,025 (92.7%)	26,753 (93.5%)	45,778 (93.2%)
**Chronic bronchitis**			
Yes	695 (4.4%)	882 (3.9%)	1,577 (4.1%)
No	15,167 (95.6%)	21,518 (96.1%)	36,685 (95.9%)
**Asthma**			
Yes	815 (5.1%)	1,434 (6.4%)	2,249 (5.9%)
No	15,050 (94.9%)	20,974 (93.6%)	36,024 (94.1%)
**High blood cholesterol**			
Yes	6,763 (42.7%)	8,619 (38.5%)	15,382 (40.3%)
No	9,081 (57.3%)	13,749 (61.5%)	22,830 (59.7%)
**Has been vaccinated against COVID-19**			
No	3,603 (17.5%)	5,784 (20.2%)	9,387 (19.1%)
Yes	16,942 (82.5%)	22,843 (79.8%)	39,785 (80.9%)
**Has been vaccinated against influenza**			
No	12,292 (59.9%)	17,803 (62.3%)	30,095 (61.3%)
Yes	8,218 (40.1%)	10,770 (37.7%)	18,988 (38.7%)

Figure 1 shows that vaccination coverage for COVID-19 was over 90% in Germany, Sweden, Netherlands, Spain, Italy, Denmark, Belgium, Portugal, Finland, and Malta, with the highest percentage being in Denmark (98.2%) and Malta (98.2%). The countries where less than 50% vaccination coverage included Bulgaria (19.1%) and Romania (32.7%). Vaccination coverage for influenza was comparatively lower for all the countries. The countries with the highest vaccination coverage for influenza included Malta (66.7%), Spain (63.7%) and the Netherlands (62.5%), and those with the lowest coverage included Bulgaria (3.7%), Slovakia (5.8%) and Poland (9.2%).

**Fig. 2:**
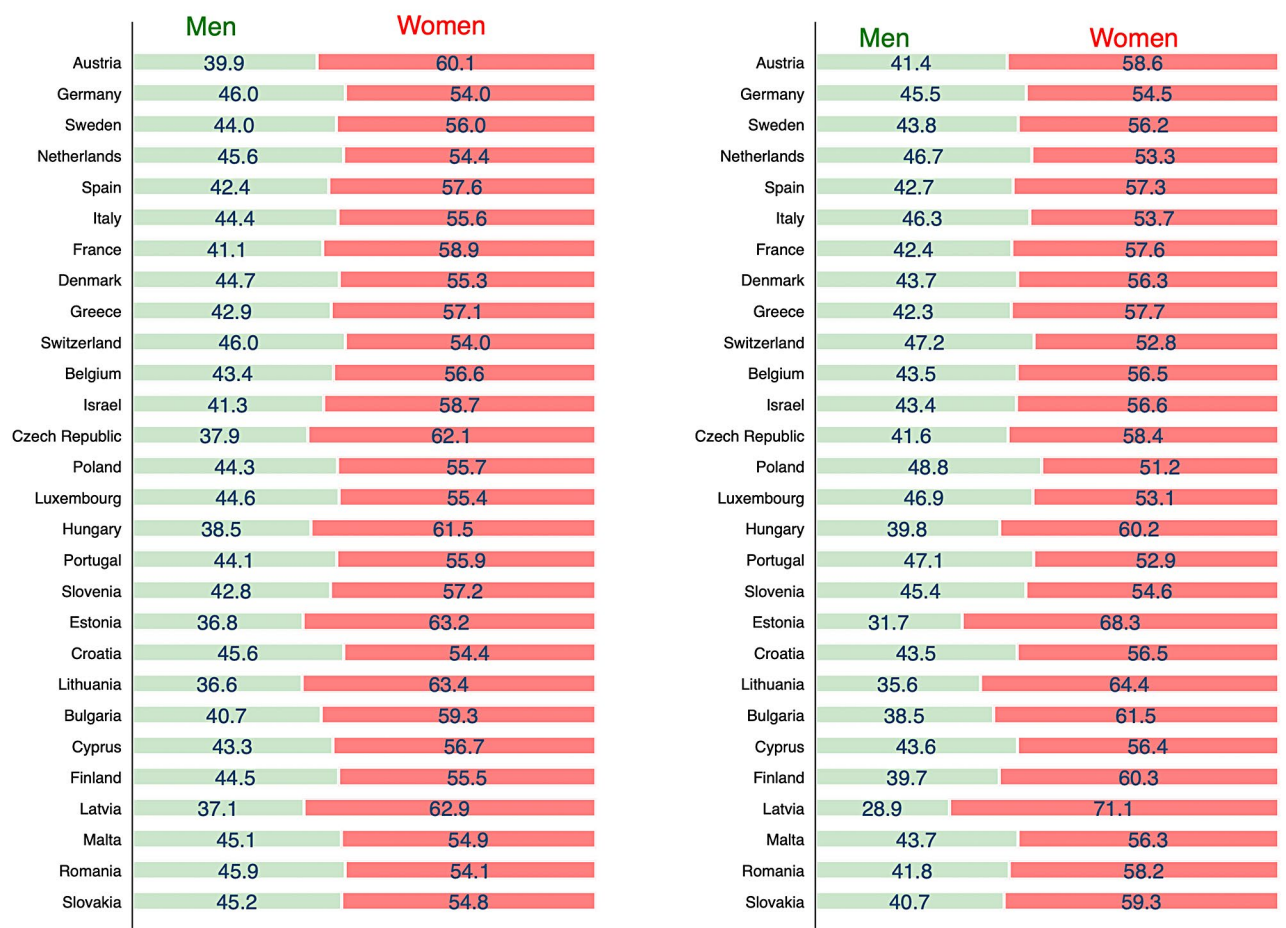
Percentage of men and women who took COVID-19 and influenza vaccines by country

Figure 2 illustrates that COVID-19 (left) and influenza (right) vaccination coverage was relatively higher among women than men. The largest male-female difference in COVID-19 vaccination was noted in Lithuania (36.6% among men vs. 63.4% among women) and Estonia (36.8% among men vs. 63.2% among women), and that for influenza vaccination in Latvia (28.9% among men vs. 71.1% among women) and Estonia (31.7% among men vs. 68.3% among women).

The results of the association between COVID-19 vaccination and self-reported status of chronic conditions are presented in Table 2. Participants with high blood pressure were 3% less likely [Risk difference = -0.03, 95% confidence interval = -0.04, -0.03], with chronic lung disease 4% less likely [Risk difference = -0.04, 95% confidence interval = -0.06, -0.03] and with chronic bronchitis were 3% less likely [Risk difference = 0.03, 95% confidence interval = -0.05, -0.01] to report taking COVID-19 vaccine. After stratification by sex, the negative effect of these three conditions was found to be more prominent among women than men. The table further illustrates that participant with high blood cholesterol [Risk difference = 0.05, 95% confidence interval = 0.05,0.06] were more likely to report taking the taking COVID-19 vaccine than participants without those conditions.

**Table 2:** Risk difference of COVID-19 vaccination coverage among men and women in relation to self-reported chronic conditions

On medication for	Full sample	Men	Women
High blood pressure (HBP)	-0.03^***^ [-0.04, -0.03]	-0.02^**^ [-0.03, -0.01]	-0.04^***^ [-0.06, -0.03]
High blood cholesterol	0.05^***^ [0.05,0.06]	0.06^***^ [0.05,0.07]	0.05^***^ [0.04,0.06]
Chronic lung disease	-0.04^***^ [-0.06, -0.03]	-0.02^*^ [-0.03, -0.01]	-0.08^***^ [-0.09, -0.06]
Diabetes	-0.01 [-0.02, 0.01]	0.01 [-0.01,0.02]	-0.02^**^ [-0.04, -0.01]
Chronic bronchitis	-0.03^*^ [-0.05, -0.01]	-0.03 [-0.06, -0.01]	-0.03^*^ [-0.06, -0.01]
Asthma	-0.01 [-0.02,0.02]	0.01 [-0.02,0.04]	0.01 [-0.02,0.03]

N.B. Risk difference with 95% confidence intervals in brackets. All regression models are adjusted for age, sex, employment and self-reported health status. Level of significance: ^*^p < 0.05, ^**^p < 0.01, ^***^p < 0.001

The results of the association between influenza vaccination and self-reported status of chronic conditions are presented in Table 3. Participants with high blood pressure were 3% less likely [Risk difference = -0.03, 95% confidence interval = -0.04, -0.01] and those with chronic lung disease were 2% less likely [Risk difference = -0.02, 95% confidence interval = -0.04, -0.01] to report taking influenza vaccine during the last 12 months. The table further illustrates that participants with high blood cholesterol [Risk difference = 0.11, 95% confidence interval = 0.10,0.12], chronic bronchitis [Risk difference = 0.06, 95% confidence interval = 0.03,0.09] and asthma [Risk difference = 0.05, 95% confidence interval = 0.02,0.07] were more likely to report taking the influenza vaccine than participants without those conditions.

**Table 3:** Risk difference of influenza vaccination coverage among men and women in relation to self-reported chronic conditions

	Full sample	Men	Women
HBP	-0.03^***^ [-0.04, -0.01]	-0.01 [-0.03, 0.00]	-0.03^***^ [-0.05, -0.02]
High blood cholesterol	0.11^***^ [0.10,0.12]	0.11^***^ [0.10,0.13]	0.11^***^ [0.09,0.12]
Chronic lung disease	-0.02^**^ [-0.04, -0.01]	0.01 [-0.02,0.03]	-0.05^***^ [-0.07, -0.03]
Diabetes	-0.01 [-0.02,0.01]	0.01 [-0.01,0.03]	-0.01 [-0.03,0.01]
Chronic bronchitis	0.06^***^ [0.03,0.09]	0.08^***^ [0.04,0.12]	0.03 [-0.01,0.07]
Asthma	0.05^***^ [0.02,0.07]	0.05^*^ [0.01,0.09]	0.05^**^ [0.02,0.09]

N.B. Risk difference with 95% confidence intervals in brackets. All regression models are adjusted for age, sex, employment and self-reported health status. Level of significance: ^*^p < 0.05, ^**^p < 0.01, ^***^p < 0.001

The results of the association between taking both COVID-19 and influenza vaccination and self-reported status of chronic conditions are presented in Table 4. Men and women with high blood pressure were 3% less likely to have reported taking both of the vaccines. Women with chronic lung disease were 5% less likely [Risk difference = -0.05, 95% confidence interval = -0.06, -0.03] to have reported taking both of the vaccines. In contrast, the likelihood of reporting the uptake of both of the vaccines was higher among those with high blood cholesterol [Risk difference = 0.11, 95% confidence interval = 0.10,0.12], chronic bronchitis [Risk difference = 0.05, 95% confidence interval = 0.02,0.08] and asthma [Risk difference = 0.06, 95% confidence interval = 0.03,0.08].

**Table 4:** Risk difference of taking both COVID-19 and flu vaccination

	Full sample	Men	Women
HBP	-0.03^***^ [-0.04, -0.02]	-0.03^***^ [-0.05, -0.01]	-0.03^***^ [-0.04, -0.01]
High blood cholesterol	0.11^***^ [0.10,0.12]	0.12^***^ [0.10,0.13]	0.10^***^ [0.09,0.12]
Chronic lung disease	-0.01 [-0.02,0.00]	0.03^**^ [0.01,0.04]	-0.05^***^ [-0.06, -0.03]
Diabetes	0.01 [0.00-,0.02]	0.02^*^ [0.00,0.04]	0.01 [-0.02,0.02]
Chronic bronchitis	0.05^***^ [0.02,0.08]	0.08^***^ [0.04,0.12]	0.03 [-0.01,0.06]
Asthma	0.06^***^ [0.03,0.08]	0.05^**^ [0.02,0.09]	0.07^***^ [0.04,0.09]

N.B. Risk difference with 95% confidence intervals in brackets. All regression models are adjusted for age, sex, employment and self-reported health status. Level of significance: ^*^p < 0.05, ^**^p < 0.01, ^***^p < 0.001

## Discussion

The dual pandemic of Covid-19 and influenza is having an increasing impact on adult individuals with chronic diseases. This public health issue has caused a great deal of concern among medical professionals, as the combination of these two illnesses could be particularly detrimental to older individuals living with preexisting conditions. Hence, it is essential to understand how this double pandemic is affecting countries with a relatively larger proportion of older population in order to ensure that appropriate preventative measures are being taken. In light of this, the present study aimed to measure the correlation between the uptake of COVID-19 and influenza vaccines and non-communicable chronic disease status among adult men and women in the European Union. The descriptive findings indicate that about four-fifth of the participants reported taking COVID-19, and about two-fifth (39%) reported taking the influenza vaccine during the last 12 months. The percentage varied considerably at the country level, ranging from over 90% in Germany, Sweden, Netherlands, Spain, Italy, Denmark, Belgium, Portugal, Finland, and Malta to less than 50% in Bulgaria and Romania. The causes of non-uptake of vaccines were not collected in the SHARE survey, however, another EU-based multi-country study reported that fear of side effects constituted the most commonly cited cause of vaccine hesitancy (from 22% of in Spain to 41% in Italy). [26].

Of note, the percentage of participants who reported taking the influenza vaccination was comparatively lower for all the countries. In addition to the intercountry differences, there was also a noticeable male-female gap in the percentage of both COVID-19 and influenza vaccination, with the percentage being relatively higher among women than men. The largest male-female difference in COVID-19 vaccination was noted in Lithuania and Estonia, and that for influenza vaccination in Latvia and Estonia. The male-female gap in vaccination status has been highlighted in previous studies as well. The exact causes behind the differences are hard to pinpoint, as vaccination behavior can be shaped by various environmental, socioeconomic, and healthcare-related factors. One explanation might be the ratio of older women compared to older men in nearly all populations globally. [27] Women also occupy a larger proportion of workforce in the service sector, including healthcare and education that are more likely to require being fully vaccinated. [28] Regardless of the causes, the lower vaccination rate among men pose a significant challenge for healthcare systems as the risk of infection and other COVID-19 related health outcomes are comparatively worse among men than among women. [29, 30].

Regarding the association between vaccination and the preexisting chronic conditions, we found that participants with high blood pressure, chronic bronchitis and chronic lung disease were less likely to report taking COVID-19 vaccine. In contrast, participants with high blood cholesterol were more likely to report taking the COVID-19 vaccine than participants without those conditions. The likelihood of taking influenza vaccine was also lower among participants with high blood pressure, but lower among those with high blood cholesterol, chronic bronchitis and asthma. Of the six preexisting conditions, only high blood pressure and chronic lung disease were found be consistently associated with lower likelihood of taking both of the vaccines. However, with regard to the likelihood of taking both of the vaccines, only high blood pressure was found to be inversely associated but not chronic lung disease. In the sex-stratified models, the likelihood of not taking each of the vaccine was relatively lower among diabetic and hypertensive women compared to their male counterparts. In short, the current findings reveal a dissimilar vaccination pattern among individuals in different disease groups, with high blood pressure and chronic lung disease showing a consistently negative association with the uptake of both of the vaccines. The underlying mechanism behind this difference is hard to decode from the current analysis, and therefore offers opportunities for further research. Vaccines are an important tool in preventing disease and promoting public health, yet the present analysis uncovers notable disparities in the rates of vaccination among countries, and between men and women in the EU. Vaccination is particularly important for protecting vulnerable populations living with chronic diseases, and understanding why some disease groups may be less vaccinated than others can help public health programs to better target preventive measures.

This study has several important limitations to consider as well. Firstly, the data used in this analysis are cross-sectional and therefore, no causal relationship can be established between the outcome and explanatory variables. Since the analysis was based on pre-existing data, the authors had no control over the selection and measurement of the study variables e.g. measurement of the diseases or vaccination status. The list of chronic illnesses was also not exhaustive and includes only a limited number of diseases. The inclusion of a small number of explanatory variables may leave room for unmeasured confounding and potentially influence the observed associations. The analysis cannot clarify the nature or quality of the relationships as well. The results should be also be interpreted in light the possible reporting bias and the large differences in sample size between countries. Another important limitation was that there were no healthcare system related factors such as availability of vaccines, awareness of COVID-19 vaccine, or distance to a health facility. There was also no data on the causes of non-uptake of vaccines. A cross-sectional study among elderly individuals in Hong Kong reported that the main reasons cited for non-uptake of COVID-19 vaccines were “Not feeling in good health” (27%), “Worry about vaccine side effects” (18%), “Feeling no need” (10%), and “Lack of recommendation from doctors” (9%). [31] Regardless of these limitations, these findings contribute to the literature on the difference in vaccination patterns among individuals with different preexisting conditions.

## Conclusion

In conclusion, the present study identified significant regional and sex disparities in the uptake of COVID-19 and influenza vaccination across the study countries. The results suggest that individuals with preexisting medical conditions, such as high blood pressure and chronic lung disease, are less likely to take the vaccines, whereas those with high blood cholesterol are more likely to receive them. Furthermore, participants with high blood pressure and high blood cholesterol were less likely to receive the influenza vaccine compared to those with chronic bronchitis and asthma. Finally, only high blood pressure and chronic lung illness showed consistent associations with reduced vaccination rates for both COVID-19 and influenza among the six preexisting diseases examined. Given the persistent disparities in vaccine uptake observed among different risk groups, further epidemiological studies are needed to gain a better understanding of the factors contributing to vaccination behavior and to identify effective strategies to address them.

### Electronic supplementary material

Below is the link to the electronic supplementary material.

Supplementary Material 1: Supplementary figures and tables

## Data Availability

Data are available from the Survey of Health, Ageing and Retirement in Europe website upon registration: https://share-eric.eu/.
