# Supplementary material for: Uptake of COVID-19 and influenza vaccines in relation to preexisting chronic conditions in the European countries
Source: BMC Geriatr. 2024 Jan 12;24:56. doi: 10.1186/s12877-023-04623-5 (PMC10785450; doi:10.1186/s12877-023-04623-5)
Supplement: Supplementary file 1 — Supplementary Material 1: Supplementary figures and tables [file 12877_2023_4623_MOESM1_ESM.docx]

Figure 1a: Sample size by country:


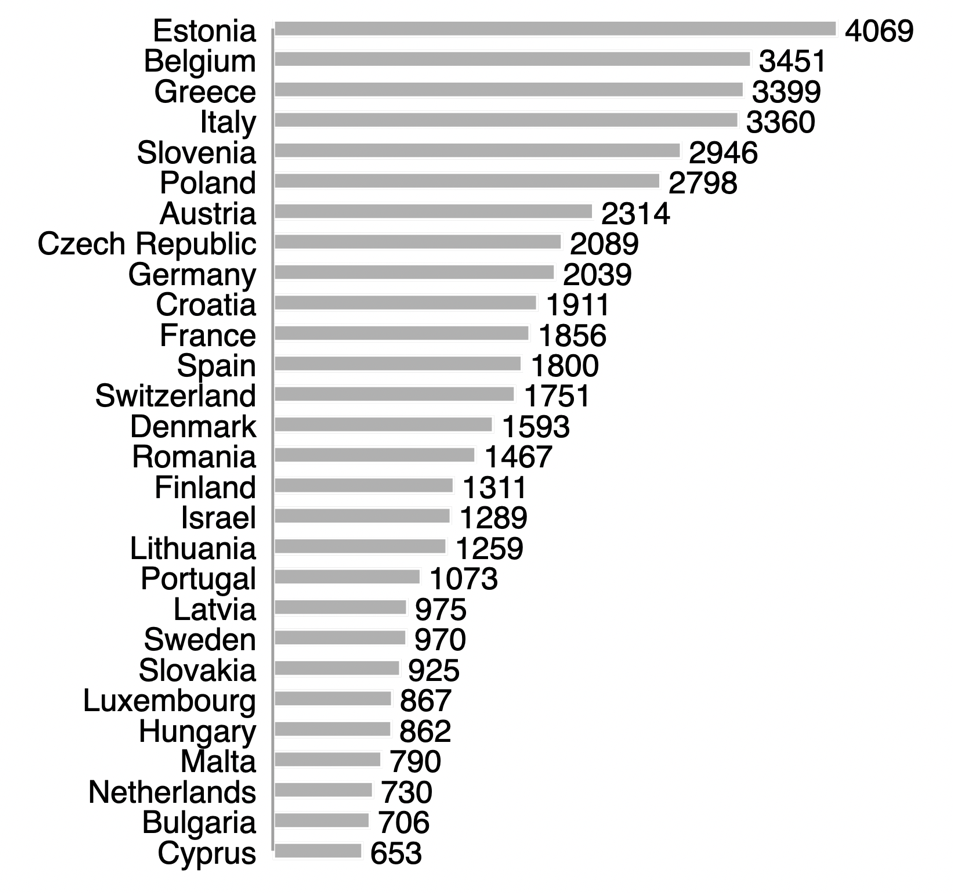


Table 1a: odds ratios of COVID-19 vaccination coverage among men and women in relation to self-reported chronic conditions.

| On medication for | **Full sample** | **Men** | **Women** |
| --- | --- | --- | --- |
| High blood pressure (HBP) | 1.62^***^ [1.53,1.72] | 1.81^***^ [1.64,1.99] | 1.51^***^ [1.40,1.62] |
|  |  |  |  |
| High blood cholesterol | 0.74^***^ [0.70,0.79] | 0.80^***^ [0.73,0.88] | 0.72^***^ [0.67,0.78] |
|  |  |  |  |
| Chronic lung disease | 0.73^***^ [0.68,0.78] | 0.87^*^ [0.78,0.97] | 0.63^***^ [0.57,0.68] |
|  |  |  |  |
| Diabetes | 0.98 [0.92,1.05] | 1.10 [0.99,1.23] | 0.89^*^ [0.82,0.98] |
|  |  |  |  |
| Chronic bronchitis | 0.80^**^ [0.70,0.92] | 0.85 [0.67,1.06] | 0.77^**^ [0.64,0.92] |
| Asthma | 1.08 [0.96,1.23] | 1.09 [0.87,1.36] | 1.13 [0.97,1.31] |

Table 2a: odds ratios of influenza vaccination coverage among men and women in relation to self-reported chronic conditions.

| On medication for | **Full sample** | **Men** | **Women** |
| --- | --- | --- | --- |
| High blood pressure (HBP) | 1.58^***^ [1.52,1.65] | 1.60^***^ [1.49,1.70] | 1.57^***^ [1.48,1.66] |
|  |  |  |  |
| High blood cholesterol | 0.90^***^ [0.87,0.94] | 0.89^**^ [0.84,0.96] | 0.92^**^ [0.87,0.97] |
|  |  |  |  |
| Chronic lung disease | 0.97 [0.92,1.02] | 1.10^*^ [1.02,1.19] | 0.84^***^ [0.78,0.91] |
|  |  |  |  |
| Diabetes | 1.05 [1.00,1.10] | 1.08^*^ [1.00,1.17] | 1.01 [0.94,1.08] |
|  |  |  |  |
| Chronic bronchitis | 1.31^***^ [1.18,1.46] | 1.45^***^ [1.23,1.71] | 1.21^*^ [1.05,1.40] |
| Asthma | 1.27^***^ [1.16,1.39] | 1.24^**^ [1.06,1.45] | 1.32^***^ [1.17,1.48] |

Table 3a: odds ratios of both COVID-19 and influenza vaccination coverage among men and women in relation to self-reported chronic conditions.

| On medication for | **Full sample** | **Men** | **Women** |
| --- | --- | --- | --- |
| High blood pressure (HBP) | 1.58^***^ [1.51,1.65] | 1.62^***^ [1.51,1.73] | 1.54^***^ [1.45,1.63] |
|  |  |  |  |
| High blood cholesterol | 0.88^***^ [0.85,0.92] | 0.88^***^ [0.83,0.94] | 0.89^***^ [0.84,0.94] |
|  |  |  |  |
| Chronic lung disease | 0.96 [0.91,1.01] | 1.11^**^ [1.03,1.20] | 0.82^***^ [0.76,0.88] |
|  |  |  |  |
| Diabetes | 1.05 [1.00,1.10] | 1.09^*^ [1.01,1.18] | 1.00 [0.93,1.07] |
|  |  |  |  |
| Chronic bronchitis | 1.23^***^ [1.10,1.37] | 1.37^***^ [1.16,1.61] | 1.13 [0.97,1.30] |
| Asthma | 1.27^***^ [1.15,1.39] | 1.25^**^ [1.07,1.46] | 1.31^***^ [1.17,1.47] |

Table 4a: Interaction between sex and the chronic conditions COVID-19 vaccination coverage

|  | HBP | High blood cholesterol | Chronic lung disease | Diabetes | Chronic bronchitis | Asthma |
| --- | --- | --- | --- | --- | --- | --- |
| No # Male | ref | ref | ref | ref | ref | ref |
| Yes # Male | 1.77^***^ [1.61,1.95] | 1.32^***^ [1.23,1.42] | 0.95 [0.86,1.06] | 1.51^***^ [1.37,1.67] | 0.88 [0.72,1.09] | 1.02 [0.83,1.25] |
|  |  |  |  |  |  |  |
| Yes # Female | 1.09^*^ [1.01,1.18] | 0.93^*^ [0.87,0.99] | 0.50^***^ [0.46,0.55] | 0.90^*^ [0.83,0.98] | 0.57^***^ [0.49,0.68] | 0.74^***^ [0.64,0.85] |
|  |  |  |  |  |  |  |
| No # Female | 0.82^***^ [0.77,0.88] | 1.00 [0.93,1.06] | 0.79^***^ [0.74,0.84] | 0.90^***^ [0.86,0.95] | 0.74^***^ [0.70,0.78] | 0.73^***^ [0.69,0.78] |
|  |  |  |  |  |  |  |

Odds ratios with 95% confidence intervals in brackets

^*^ *p* < 0.05, ^**^ *p* < 0.01, ^***^ *p* < 0.001

Table 5a: Interaction between sex and the chronic conditions influenza vaccination coverage

|  | HBP | High blood cholesterol | Chronic lung disease | Diabetes | Chronic bronchitis | Asthma |
| --- | --- | --- | --- | --- | --- | --- |
| No # Male | ref | ref | ref | ref | ref | ref |
| Yes # Male | 1.63^***^ [1.53,1.73] | 1.40^***^ [1.32,1.48] | 1.21^***^ [1.12,1.30] | 1.51^***^ [1.41,1.62] | 1.59^***^ [1.36,1.85] | 1.40^***^ [1.21,1.61] |
|  |  |  |  |  |  |  |
| Yes # Female | 1.36^***^ [1.28,1.44] | 1.21^***^ [1.15,1.28] | 0.82^***^ [0.76,0.88] | 1.22^***^ [1.14,1.30] | 1.17^*^ [1.02,1.34] | 1.16^**^ [1.04,1.29] |
|  |  |  |  |  |  |  |
| No # Female | 0.89^***^ [0.84,0.94] | 0.95 [0.90,1.00] | 0.90^***^ [0.86,0.94] | 0.94^**^ [0.90,0.98] | 0.86^***^ [0.82,0.90] | 0.85^***^ [0.81,0.89] |
|  |  |  |  |  |  |  |

Table 6a: Interaction between sex and the chronic conditions for taking both COVID-19 and influenza vaccination coverage

|  | HBP | High blood cholesterol | Chronic lung disease | Diabetes | Chronic bronchitis | Asthma |
| --- | --- | --- | --- | --- | --- | --- |
| No # Male | ref | ref | ref | ref | ref | ref |
| Yes # Male | 1.65^***^ [1.55,1.76] | 1.38^***^ [1.31,1.46] | 1.22^***^ [1.13,1.31] | 1.52^***^ [1.41,1.63] | 1.50^***^ [1.29,1.75] | 1.38^***^ [1.20,1.59] |
|  |  |  |  |  |  |  |
| Yes # Female | 1.31^***^ [1.23,1.39] | 1.15^***^ [1.09,1.21] | 0.77^***^ [0.71,0.83] | 1.16^***^ [1.09,1.24] | 1.05 [0.92,1.21] | 1.10 [0.98,1.22] |
|  |  |  |  |  |  |  |
| No # Female | 0.88^***^ [0.83,0.93] | 0.94^*^ [0.89,0.99] | 0.88^***^ [0.84,0.93] | 0.92^***^ [0.88,0.96] | 0.84^***^ [0.80,0.87] | 0.83^***^ [0.79,0.86] |
|  |  |  |  |  |  |  |
